# Metabolomic Approaches to Lung Function in Pediatric Asthma: A Narrative Review

**DOI:** 10.3390/children13040544

**Published:** 2026-04-14

**Authors:** Orlanda Moldovan, Paraschiva Cherecheș-Panța, Valentina Sas, Robert Simon, Sorin Claudiu Man

**Affiliations:** 1 Doctoral School, Iuliu Hațieganu University of Medicine and Pharmacy, 400012 Cluj-Napoca, Romania; orlanda.simon@umfcluj.ro (O.M.);; 2 Third Pediatric Discipline, Mother and Child Department, Faculty of Medicine, Iuliu Hațieganu University of Medicine and Pharmacy, 400132 Cluj-Napoca, Romania; 3 Third Pediatric Clinic, Clinical Hospital for Pediatric Emergencies, 400132 Cluj-Napoca, Romania; 4 Anesthesia and Intensive Care Department, Faculty of Medicine, Iuliu Hatieganu University of Medicine and Pharmacy, 400012 Cluj-Napoca, Romania; robert.simon@umfcluj.ro; 5 Clinical Institute of Urology and Renal Transplantation, 400006 Cluj-Napoca, Romania

**Keywords:** pediatric asthma, metabolomics, breathomics, volatile organic compounds, exhaled breath condensate, lung function

## Abstract

**Highlights:**

**What are the main findings?**

- Metabolomic studies in pediatric asthma identify alterations in lipid, sphingolipid, purine, and oxidative stress pathways that are associated with airflow limitation and asthma endotypes.
- Breathomic analysis of volatile organic compounds and exhaled breath condensate demonstrates associations with lung function parameters, including FEV1 and small airway indices.

**What are the implications of the main findings?**

- Metabolomic and breathomic profiling may complement conventional lung function testing by providing additional insight into airway inflammation, airflow impairment, metabolic dysregulation, and disease heterogeneity.
- Further standardization, larger pediatric cohorts, longitudinal validation studies, and integration with complementary multi-omics data are required to clarify clinical utility before routine implementation.

**Abstract:**

**Introduction**: Asthma is one of the most common chronic diseases in childhood and represents a major global public health concern due to its high prevalence, healthcare burden, and impact on quality of life. Pediatric asthma is characterized by clinical and biological heterogeneity, reflected in variable airflow limitations and distinct inflammatory endotypes. Conventional diagnostic tools do not fully capture the metabolic mechanisms underlying lung function impairment and disease variability. **Aim**: This narrative review aims to synthesize evidence published linking metabolomic and breathomic signatures to lung function parameters in children with asthma. **Methods**: We searched PubMed, Scopus, and Google Scholar using predefined keywords including pediatric asthma, metabolomics, breathomics, volatile organic compounds, exhaled breath condensate, and lung function. The search covered publications from January 2015 to January 2026. Earlier studies were included when necessary for the conceptual or methodological context. We included human studies evaluating metabolomic or breathomic profiles in children (≤18 years) and reporting associations with lung function, severity, endotypes, or exacerbations. Duplicate records, adult-only studies, animal models, non-English publications, and conference abstracts without full data were excluded. **Results**: Alterations in lipid and sphingolipid metabolism, oxidative stress pathways, and purine metabolism were associated with airflow limitation and reduced FEV1. Breathomic analyses revealed associations between volatile profiles, small airway dysfunction, and inflammatory patterns. However, findings remain heterogeneous across biological matrices and analytical platforms. **Conclusions**: Metabolomic and breathomic profiling may complement conventional lung function assessment by providing additional mechanistic insight into pediatric asthma heterogeneity. Standardized methodologies, longitudinal validation, and integration within multi-omics approaches are required before routine clinical implementation.

## 1. Introduction

Asthma is a heterogeneous disease characterized by chronic airway inflammation, with symptoms such as coughing, wheezing, dyspnea, and chest tightness that may vary over time and in intensity. A variable degree of expiratory flow restriction is present [1]. Asthma is the most common chronic respiratory disease in children, with the highest prevalence in the 5–9-year age group, predominantly among males [2,3,4].

Globally, approximately 9% of children are affected [5,6], and although the standardized prevalence has declined slightly in recent decades, the disease’s incidence remains high and is estimated to remain elevated until 2050. Caring for pediatric asthma patients is often difficult due to early onset, continuous exposure to risk factors, and unequal access to medical services. In regions with low socioeconomic status, although prevalence is lower, asthma-related mortality and disability are significantly higher, reflecting treatment limitations and the economic impact on families [6].

The aim of this narrative review is to synthesize current evidence linking metabolomic and breathomic signatures with lung function parameters in pediatric asthma. This is clinically relevant, as small airway dysfunction, lung function decline, exacerbation risk, and asthma endotypes may not be fully captured by conventional diagnostic tools. Despite growing research interest, important gaps remain, including limited standardization of VOC and exhaled breath condensate methodologies, insufficient longitudinal validation, and methodological variability. This review maps findings across biological matrices, analytical platforms, and functional respiratory indices to clarify current evidence and its translational potential.

To synthesize the current landscape of metabolomic and breathomic applications in pediatric asthma, we conducted a systematic literature search across PubMed, Scopus, and Google Scholar databases. The search was restricted to human studies published between January 2015 and January 2026, focusing on pediatric cohorts aged 18 years or younger. The search strategy employed a combination of keywords such as “pediatric asthma,” “metabolomics,” “breathomics,” “volatile organic compounds (VOCs),” “exhaled breath condensate (EBC),” and “lung function”. Studies were included if they provided primary data linking metabolic signatures—derived from blood, urine, or exhaled breath—to objective respiratory indices such as FEV1, FEF25–75%, or airway hyper-responsiveness. Articles focusing exclusively on adult populations, animal models, conference abstracts without full data or those published in languages other than English were excluded to ensure the relevance and consistency of the findings.

## 2. Asthma Phenotypes and Endotypes

A traditional, oversimplified classification divides asthma into two broad categories: extrinsic (allergic) asthma, triggered by allergens and characterized by type 2 T helper (Th2) inflammation, and intrinsic asthma, triggered by several factors, including exposure to cold, infections, physical exertion, obesity, and strong emotions [7].

Clinically, the disease is characterized by several distinct phenotypes: allergic asthma, non-allergic asthma (eg. viral-induced asthma), asthma with variable or predominant cough, asthma with adult-onset, and asthma associated with obesity [1].

From a pathophysiological point of view, asthma is divided into two endotypes: Th2-high (with eosinophilic airway inflammation), mediated by cytokines such as IL-4, IL-5, IL-13, and Th2-low (with neutrophilic or paucigranulocytic inflammation), which can be mediated by cytokines such as IL-17, TNF-α, and IFN-γ [7,8,9].

Children with severe asthma have higher median serum levels of TNF-α and IL-5 than those with non-severe asthma. Additionally, IL-17, IL-4, and IL-5 showed negative correlations with pulmonary function parameters, such as FEV1, FEV1/FVC, FEF25–75%, and FEFmax, suggesting that elevated levels of these cytokines are linked to impaired lung function. IL-17 and IL-5 were particularly associated with airway obstruction, and IL-17 was also related to bronchodilator response in FEV1 values, highlighting the role of both T2-high and T2-low immune responses in modulating airway inflammation and lung function in childhood asthma [9].

T2-high inflammation, characterized by eosinophilic airway inflammation, is more common in children and is often associated with allergic (atopic) asthma, which accounts for approximately 80% of pediatric asthma cases. By contrast, T2-low inflammation is more common in adults and is associated with a weaker response to corticosteroids [3,7].

There are a few studies that focus exclusively on phenotyping asthma in children, some with mixed cohorts that include children, and others on children, but in small patient groups [10].

The biomarkers currently used in practice for Th2-high asthma include sputum eosinophils, FeNO (fractional exhaled nitric oxide), and eosinophil blood count. Sputum eosinophils > 3% indicate eosinophilic inflammation in the airways. Blood eosinophil count is a more accessible method, but it is less specific because it can also be elevated in other allergic diseases. High blood eosinophil counts, together with elevated FeNO levels, suggest Th2-type inflammation [1,7]. Other important biomarkers include serum IgE, aeroallergen sensitization panel, and serum periostin [8]. Measurement of Th2 cytokines (IL-4, IL-5, IL-13, and TSLP) is primarily used in research [7].

The Th2-low asthma endotype is characterized by the absence of eosinophilic inflammation and low FeNO, but by neutrophilic or paucigranulocytic inflammation. Available biomarkers include induced sputum neutrophils, which can reach 40–76% in asthmatic patients [7]. Other potential biomarkers could be C-reactive protein and IL-6, which indicate systemic inflammation [11].

Asthma phenotypes are not static and can evolve throughout a person’s life [8,12]. Phenotypic plasticity or phenotypic shifting can be present, and it indicates that a child may begin with an allergic phenotype and, upon transitioning to adulthood, develop additional triggers and shift to a non-allergic phenotype [3,8,10].

### Clinical Management and Therapeutic Implications

Although most children can be managed with inhaled corticosteroids (ICS) as initial treatment [3], understanding the pathophysiological mechanisms of asthma is very important for precision treatment. Currently, targeted biological therapies approved for use in the pediatric population include anti-IL-4/IL-13, anti-IL-5, anti-IL-5Rα, anti-IgE, and anti-TSLP antibodies [3,4,12]. These molecules are used for moderate-to-severe asthma and have been shown to reduce the risk of severe exacerbations and the need for corticosteroids. However, choosing the most appropriate treatment is challenging because the pathological mechanisms involved in asthma often overlap [4].

## 3. Diagnosis of Asthma in Children

Asthma is diagnosed by establishing the history and pattern of symptoms, which is often difficult because symptoms overlap with those of other respiratory conditions [1,13]. It is important to document the initial exacerbation because subsequent diagnosis can be difficult once the patient has started bronchodilator treatment [1]. There is no single definitive test for asthma. The diagnosis is made by integrating symptoms, physical examination, and objective tests that demonstrate airflow variability and reversibility [1,13].

Spirometry with bronchodilator reversibility testing is the test of choice. Spirometry is feasible from approximately age 5, or when patients can understand and cooperate. If spirometry is unavailable, another test that can indicate airflow variability and may help diagnose asthma is PEF [1,13].

In younger children under 5 and even in some older children, spirometry may be difficult to perform reliably due to the need for patient cooperation and coordinated respiratory maneuvers, highlighting the need for complementary assessment tools. If asthma is suspected, inhaled corticosteroids are used, and the child is reassessed periodically until standard objective tests can be performed [13]. Objective test alternatives, such as impulse oscillometry (IO or IOS), may be used because it does not require breath coordination or forced maneuvers [14].

Eosinophil levels from sputum, FeNO, and blood eosinophil counts are used. Although the sputum eosinophil count is considered a reference method for assessing eosinophilic airway inflammation, sputum induction is technically difficult in younger children, limiting its applicability in routine pediatric practice [3]. Increased levels of FeNO (50 ppb or higher in those 16 years or older and 35 ppb or higher in the 5–16 age group) support the diagnosis [13]. Correlations of FeNO with lung function parameters such as FEV1 are generally modest and inconsistent. This discrepancy reflects the fact that FeNO measures inflammatory activity, whereas spirometry assesses mechanical airflow limitation. Therefore, FeNO and pulmonary function tests should be considered complementary tools in the assessment of asthma [15]. Normal blood eosinophil levels cannot rule out asthma [1].

## 4. Future of Understanding and Treating Pediatric Asthma

### 4.1. The Challenges of Pediatric Asthma

Diagnosing, phenotyping, endotyping, and treating asthma remain a challenge for pediatric patients. More biomarkers are needed to characterize the pathophysiological mechanisms of asthma, improve diagnosis, and enable precision treatment [12,16].

Severe bronchiolitis in infancy has been associated with a higher prevalence of asthma and impaired lung function later in life. Without baseline functional assessment, it remains unclear whether reduced airway function was already present—suggesting an underlying predisposition—or whether bronchiolitis itself contributed to subsequent airflow impairment [17].

Current guidelines, such as GINA and NICE, have shown limitations in clinical practice. De Jong and colleagues (2020) analyzed the Swiss Paediatric Airway Cohort (SPAC), including 514 participants, to evaluate the diagnostic accuracy of the algorithms proposed by the GINA and NICE guidelines. Although 69% of participants were diagnosed with asthma, the study confirms the difficulty of establishing a definitive diagnosis in all children using current objective methods. Many patients required additional investigations, such as retesting (per GINA) or home PEF monitoring (per NICE), highlighting the limitations of standard clinical and paraclinical methods [18].

### 4.2. The Rise of Metabolomics and Multi-Omics

**Figure 1:**
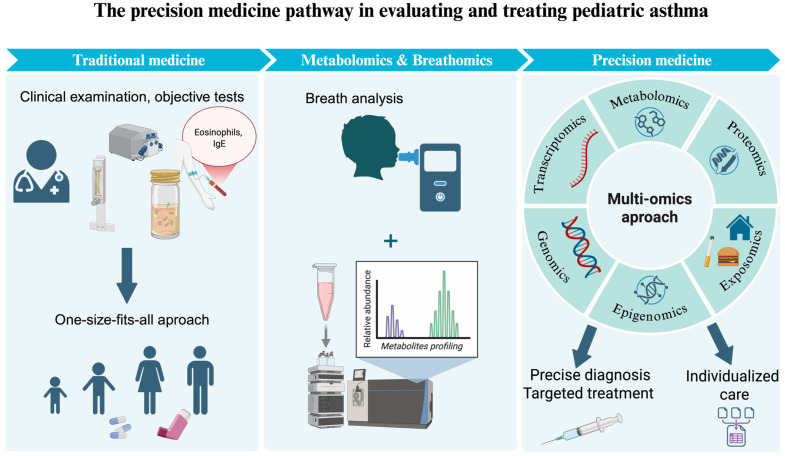
The precision medicine pathway in evaluating and treating pediatric asthma. Schematic representation of the transition from conventional evaluation (including spirometry, sputum analysis, FeNO, blood eosinophils, IgE and other inflammatory biomarkers) to integrated metabolomic and breathomic profiling (VOCs and exhaled breath condensate), and further toward multi-omics approaches to support endotype-based assessment and individualized management.

Over the past two decades, progress has been made in high-performance “omics” technologies and in the multi-omics approach to understanding the complexity of asthma [11]. As illustrated in Figure 1, this evolution reflects a fundamental transformation in asthma management, moving beyond traditional approaches toward a precision medicine model.

Several large-scale multi-omics projects aim to better understand asthma in children and adults by integrating information across multiple omics levels. These projects, such as the Severe Asthma Research Program (SARP), Unbiased BIOmarkers in PREDiction of respiratory diseases outcomes (U-BIOPRED), Mechanisms of the Development of Allergy (MeDALL) study, and Systems Pharmacology Approach to Difficult-to-treat Pediatric Asthma (SysPharmPediA), integrate multi-omics data to better understand asthma pathophysiology and phenotypes [19,20,21,22].

### 4.3. Metabolomics in Asthma

Within the omics landscape, metabolomics involves analyzing small molecules (50–1500 Da) and cellular metabolic products, as well as their ratios [12,23]. Metabolites are small molecular structures, which can be, for example, lipids, amino acids, carbohydrates, alcoholic compounds, nucleic acids, etc. [24] They can be endogenous (primary metabolites) or exogenous (secondary metabolites) [25]. If the values of these molecules fall outside reference ranges, this could indicate certain pathological conditions [23,24].

The human metabolome (the complete collection of metabolites) is difficult to quantify, mainly because the number of metabolites (to date, tens of thousands in *Homo sapiens*) is much larger than the number of genes or proteins [25]. At the same time, the devices and techniques required to separate and identify metabolome components are far more numerous than those needed to elucidate the genome or proteome [23,24].

Metabolomics employs a range of analytical approaches and techniques, each with distinct advantages and limitations. Metabolomic analysis is primarily performed using liquid or gas chromatography coupled to mass spectrometry (LC-MS/GC-MS). Another technique used is nuclear magnetic resonance spectroscopy, which has lower sensitivity and specificity than mass spectrometry [12,26,27].

These techniques enable global exploration of the metabolome and the identification of new metabolic pathways involved in complex diseases such as asthma. They generally provide relative quantification and require further validation [23,26]. Metabolomic analysis can be targeted, allowing precise quantification of predefined metabolites and making it more clinically applicable, but it is limited to preselected molecules. In contrast, untargeted metabolomics analyzes as many metabolites as possible without a hypothesis and looks for differences between groups of samples [25]. Combining both untargeted and targeted metabolomics is often necessary for a comprehensive study [27,28].

#### 4.3.1. The Gut–Lung Axis and Microbial Metabolites

The human gut microbiome is predominantly found in the colon and comprises multiple microbial species. It can also be analyzed from a metabolomic perspective [25]. Thus, a healthy microbiome can also be examined metabolomically by analyzing the metabolite profile. Disruption of the microbiome or the intestinal epithelial barrier is associated with the onset of certain pathologies, including asthma. This is often due to excess production and systemic absorption of certain inflammatory metabolites, such as uremic toxins, histamine, and tyramine [29]. The gut–lung axis is a relatively new concept that describes how the gut microbiome “communicates” with or influences lung tissue. Of particular interest are short-chain fatty acids, often produced by species of the genus *Clostridium* in the gut [25]. The three most important short-chain fatty acids are acetate, propionate, and butyrate, which are produced through the conversion of indigestible polysaccharides [30,31].

These fatty acids regulate the training, maturation, and maintenance of the immune system by binding to G protein-coupled receptors, which in turn regulate the expression of genes involved in cytokine production, cell differentiation, and apoptosis. It is believed that a lack of exposure to infectious agents or microorganisms in childhood can delay immune system maturation, thereby increasing the risk of developing allergic diseases [25]. Maternal antibiotic use can induce dysbiosis and disrupt the neonatal gut–lung axis, predisposing offspring to airway hyper-responsiveness, and may fundamentally alter lung development [32,33]. Early-life environmental and microbial exposures are thought to influence asthma susceptibility, in line with concepts associated with the hygiene hypothesis, suggesting that reduced microbial stimulation during early life may contribute to altered immune development and increased asthma risk [29,31,32].

#### 4.3.2. Nutrition and Early Life Predisposition to Asthma

Lee et al. (2019) [34] demonstrated a significant interrelationship among the intestinal metabolome, plasma metabolome, intestinal microbiome, nutrition, and asthma. The article shows that a series of intestinal metabolites is associated with asthma at age three. Although the specificity of certain metabolites for the diagnosis, stratification, or prognosis of asthma in the studied population subgroup has not been demonstrated, an inverse relationship has been observed between exclusive breastfeeding during the first 4 months of life and the appearance of intestinal metabolites associated with asthma, and a direct relationship between these metabolites and the consumption of a meat-rich diet [34].

Metabolomics helps identify metabolic profiles in newborns and young children that are predictive of asthma development later in life. For example, specific metabolites in dried blood spots, urine, or plasma have been linked to asthma risk [35,36,37,38,39,40,41,42,43,44,45], as summarized in Table 1.

#### 4.3.3. Biological Matrices and Sampling Methodologies

Biological samples suitable for metabolomic analysis can be collected as early as the neonatal period and include dried blood spots, serum, plasma, exhaled breath condensate, nasopharyngeal samples, urine, and stool [46].

Serum and plasma are the most commonly used biological samples in this context because they are readily available. Given the ease and frequency of collection, urine and urinary metabolite testing have attracted researchers’ attention [47]. In plasma/serum or urine analyses, studies have shown alterations in lipid metabolism among asthmatic patients with different phenotypes and subtypes of asthma [47,48].

Using high-resolution Nuclear Magnetic Resonance (NMR) spectroscopy and partial least-squares discriminant analysis (PLS-DA), researchers identified several metabolites in urine associated with asthma development, including dimethylamine, 1-methylnicotinamide, allantoin, and guanidoacetic acid. The study included children aged 1 to 4 years old from the Prediction of Allergies in Taiwanese Children (PATCH) birth cohort [49].

#### 4.3.4. Clinical Applications

From a metabolomic perspective, asthma is the most extensively studied obstructive bronchopulmonary pathology. Given its high global prevalence in children, early diagnosis remains an important clinical objective, although the extent to which metabolomics can contribute to this goal is still under investigation. Several studies suggest that metabolomic profiles may differ between asthma phenotypes; for example, eosinophilic asthma has been associated with alterations in lipid metabolism compared with non-eosinophilic disease [48], while allergic asthma has been linked to changes in amino acid and lipid pathways relative to healthy individuals [50]. These findings remain largely descriptive and have not yet translated into clinically applicable biomarkers [48,50].

Kelly et al. (2017) [51] analyzed 21 articles reported substantial heterogeneity in metabolomic findings across biological samples, including exhaled breath condensate, urine, and blood. While no specific metabolites for asthma have been identified, a growing body of preliminary evidence suggests that specific metabolites and metabolic pathways may be associated with the early development and clinical onset of the disease [51]. The heterogeneous results may also reflect a lack of standardization in the field and could be improved by detailed research and reporting criteria. Most of the metabolites described in the 21 studies are not specific to asthma or to an asthma phenotype and are frequently observed in other respiratory diseases [52,53,54,55]. Although their role in the development and onset of asthma cannot be ruled out, their usefulness as diagnostic or prognostic markers is questionable [56,57,58,59,60,61,62]. Significant metabolites in children with asthma include adenosine, threonine, succinate, acetate, hippurate, trans-aconitate, urocanic acid, glycine, serine, and various volatile organic compounds, which are linked to pathways such as oxidative stress, hypoxia, and immunity [52,53,54,55,56,57,58,59,60,61,62,63,64].

Regarding disease severity and control, McGeachie et al. (2015) [63] failed to demonstrate differences in metabolomic profiles between patients with controlled and uncontrolled asthma based on the number of albuterol uses in the previous week, concluding that metabolomic profiles are not currently useful for differentiating or predicting asthma control. This may also be due to the small sample size of only 20 participants. The study nevertheless suggested a possible association between altered sphingolipid metabolism and uncontrolled asthma or cellular responses to albuterol, a finding that requires further validation [63].

Integrating metabolomics and transcriptomics can provide additional insights into asthma severity and prognosis [12]. Kelly et al. (2018) [65] demonstrated the use of this integrative method in a population of children with a high asthma prevalence. Using well-established methods for classifying asthma severity, including FEV1, FEV1/FVC, bronchodilator response, and methacholine response, they described a metabolomic profile integrated with a transcriptomic assessment to examine associations with lung function. This resulted in 8 gene modules and 8 metabolite modules, of which 4 and 6, respectively, were associated with lung function. Their clinical implications remain uncertain and require confirmation in larger and standardized studies [65].

### 4.4. Breath Analysis and Lung Function

While systemic metabolomics provides a holistic view of the patient’s metabolic state, breath analysis offers a direct window into the lungs’ metabolic environment. In asthma and other inflammatory obstructive pulmonary diseases, respiratory biomarkers can be assessed non-invasively through exhaled breath analysis, including VOCs and metabolites in exhaled breath condensate (EBC), both of which may be investigated using metabolomic approaches. In contrast, FeNO is a single-biomarker measurement used to assess airway inflammation, rather than a metabolomic technique. Among these methods, standardized protocols are well established for FeNO and EBC, whereas VOC analysis remains less standardized [66].

#### 4.4.1. Breathomics

Breathomics is an emerging field that analyzes VOCs with molecular weights less than 500 Da in exhaled air. Patients breathe into specialized bags (e.g., Tedlar bags) or onto adsorbent tubes that trap and pre-concentrate the gaseous compounds [27,66]. Analysis is performed using gas chromatography coupled with mass spectrometry (GC-MS) [66], electronic noses (eNoses) [27,67], or proton transfer reaction time-of-flight mass spectrometry (PTR-TOF-MS) [68].

Bajo-Fernández et al. (2014) [27] analyzed 11 studies dedicated to asthma, several of which also included pediatric populations. The analysis of VOCs in exhaled air was performed using untargeted metabolomics by GC-MS in 10 studies and, in one study, a combination of targeted and untargeted metabolomics [27]. Respiratory profiles differentiated between children with asthma and healthy children and predicted the onset of exacerbations. Although the individual compounds identified vary between studies and there is no single biomarker common to all studies, there is an overlap in chemical classes—particularly aldehydes and ketones associated with oxidative stress and airway inflammation [57,66,69,70,71,72,73,74,75,76,77].

VOC patterns can distinguish between asthma, COPD, and lung cancer [77]. In children, specific VOC signatures can predict the development of asthma as early as age 6 [73]. Certain VOCs are associated with chronic airway inflammation and can differentiate between “Th2-high” (eosinophilic) and “Th2-low” asthma [71,72]. Van Vliet et al. reported that exhaled VOC profiles were associated with clinical asthma status and small airway flow parameters (such as FEF25–75%), even in cases where FEV1 remained within normal limits [72].

VOC-based breath tests can help monitor disease control and predict exacerbations, especially in children, due to the method’s ease of use, safety, and well-tolerance [27]. Shifts in the VOC profile can predict asthma flare-ups, providing a window for preventive intervention [69]. Although it is a promising, non-invasive method, especially in children, further studies are needed to integrate it into routine medical practice [12].

Interpretation of metabolomic profiles in pediatric asthma should consider the influence of inhaled corticosteroid (ICS) treatment. Studies indicate that ICS can modulate the respiratory footprint by reducing oxidative stress-derived compounds [74]. However, the persistence of certain volatile markers despite aggressive therapy offers a unique opportunity to identify patients with treatment-resistant phenotypes, thereby facilitating the transition to biologic therapies [21,74].

Studies using eNose sensors have demonstrated that integrating the complex signals generated by the sensor array (reflecting the chemical variations in the entire VOC mixture) with machine learning algorithms enables high accuracy in discriminating between patients with severe and mild asthma. This approach is not based on identifying an individual molecule but on recognizing global breathprints specific to each stage of the disease [67,75].

#### 4.4.2. Exhaled Breath Condensate Analysis

While breathomics focuses on the direct analysis of volatile compounds in the gas phase, EBC analysis allows for the assessment of the same markers (or their derivatives) in the liquid phase [53,54]. The metabolomic analysis can be performed using Nuclear Magnetic Resonance (NMR) Spectroscopy [59,60] or liquid chromatography–tandem mass spectrometry (LC/MS), which can detect inflammatory markers such as eicosanoids and leukotrienes [53].

EBC is obtained by cooling exhaled air through contact with a cold surface (typically 0 to −20 °C), collecting samples as liquid or frozen solid material for the analysis of both volatile and non-volatile compounds. The efficiency of EBC collection depends on several factors, including the volume of air passing through the system, the condensation surface area, and the temperature gradient. To increase efficiency, especially for young children with lower expiratory flow rates, colder surfaces or exhaled-air recirculation systems can be used [65,78,79]. The technique is simple and noninvasive, posing no risk even to children with severe lung disease because it requires only normal breathing volumes. Several factors must be controlled to ensure sample quality, including breathing patterns, sampling site (large central airway versus periphery), nasal cavity contamination, and environmental factors [65,78,79,80].

In asthma, metabolomic analyses of exhaled breath condensate have shown that eicosanoids play an important role in the disease’s pathogenesis [48]. EBC contains markers of oxidative stress and inflammation, such as 8-isoprostane, leukotrienes (specifically leukotriene B4), and purines [52,53].

Metabolomic profiling of EBC can distinguish between different levels of asthma severity in children [54]. The volume of EBC collected can sometimes relate to the patient’s underlying lung volumes, though this is subject to methodological influences [78,80].

Regarding prediction, classification, and differentiation, the literature reports accuracies exceeding 85%, particularly in distinguishing subjects with asthma from healthy controls [57,58,60,62,67,76]. Differentiation between mild and severe asthma, as well as between asthma phenotypes or endotypes, has also yielded promising classification performance [54,61,64,71,75,76]. However, these findings are derived largely from small, heterogeneous cohorts and are limited by methodological variability and a lack of standardised external validation [51,66,78,79].

In asthma research, metabolomic analysis of exhaled breath has evolved beyond the identification of discriminatory biomarkers toward a more mechanistic understanding of airway disease [81,82].

VOC analysis and other exhaled biomarkers are discussed as adjuncts to conventional lung function testing, providing complementary information on airway inflammation and metabolic processes rather than direct assessment of airflow limitation [66].

Table 2 presents studies that have explored the potential of VOCs and EBC analysis in children, identifying diagnostic biomarkers, distinguishing inflammatory endotypes, and correlating with lung function.

## 5. Complementary Metabolomic Analysis Techniques

In recent years, Surface-Enhanced Raman Scattering (SERS) has been proposed as a complementary approach to classical metabolomics due to its high sensitivity, low sample requirements, and short analysis times. SERS enables rapid “metabolic fingerprinting” of biological fluids, such as serum or plasma, making it promising for screening and diagnostic applications. However, individual metabolite identification and methodological standardization remain significant limitations. Thus, integrating classical metabolomics techniques with emerging spectroscopic methods, such as SERS, may provide a more complete characterization of metabolic changes associated with allergic diseases [83].

The SERS technique is used for molecular detection and structural identification, even at very low concentrations, down to the level of a single molecule [84,85].

Raman scattering arises from inelastic collisions between photons and molecules. In these collisions, energy is exchanged, and photons can gain or lose energy to molecules. This results in the appearance of “scattered” photons with different frequencies, producing the Rama spectrum. Classical Raman spectroscopy is considered a tool for structural evaluation rather than for detecting molecular residues, including a single molecule. When spectroscopic effects occur near a metal surface or nanostructures, they are strongly influenced by coupling with surface plasmons. This leads to amplification of Raman signals from molecules near metal nanostructures, greatly increasing the potential of SERS in biomedical spectroscopy [84].

Multiple biomedical applications of SERS techniques have been described in the literature, using both direct and indirect protocols. In direct SERS, the target molecule is adsorbed onto the surface of the metallic material, and the resulting spectrum arises directly from the vibrations of the target molecule. In indirect SERS, it is necessary to attach a reporter molecule to the target molecule. Thus, detection of the reporter molecule will confirm the presence of the target molecule. Experiments using a direct protocol were conducted to detect amino acids, peptides, purines, proteins, DNA, RNA, drugs, etc., with good results, including the detection of a single molecule [85]. To study intracellular composition and dynamics, as well as the intracellular environment, colloidal gold and silver nanoparticles were used as sensors. Indirect detection protocols using SERS, which involve attaching a reporter molecule to the target molecule, have also been proposed for detecting DNA, cellular functions, and certain proteins [86].

Dynamic observation of Raman spectral changes can help characterize the ever-changing intracellular microclimate. Compared with other techniques, this approach can provide additional information about chemical properties, such as local pH [86,87].

## 6. Conclusions

Metabolomic analysis has yielded promising insights into pediatric asthma, with significant advances over the last two decades. Its clinical application to better characterize asthma endotypes and establish accessible biomarkers for more precise classification and treatment requires a cautious approach and rigorous validation through standardized, longitudinal studies.

The clinical application of metabolomic analysis in asthma management remains largely investigational, with current evidence supporting its role primarily in improving disease characterization rather than guiding routine clinical decisions. Although metabolic alterations, particularly in lipid and amino acid pathways, have been associated with distinct asthma patterns and corticosteroid responsiveness, issues related to reproducibility, cohort variability, and lack of methodological standardization limit their clinical translation. The integration of metabolomics with other omics approaches may enhance understanding of asthma heterogeneity, but challenges related to data complexity, cost, and scalability remain significant. Similarly, technologies such as electronic noses and volatile organic compound profiling show potential as non-invasive monitoring tools, yet their clinical validity and added value over established assessments require further validation.

## Figures and Tables

**Table 1:** Metabolites linked to asthma risk.

Study	Sample	Metabolites/Metabolic Signature
Brustad et al. (2023) [35]	Dried blood spot (2–3 days after birth)	Caffeine-related compounds, tryptophan betaine, stachydrine, ergothioneine
Carraro et al. (2021) [37]	Urine (at birth)	Reduced purine and amino acid metabolites (xanthine, uric acid, leucine, tyrosine, pyroglutamic acid, ornithine)
Chawes et al. (2019) [38]	Urine (4 weeks of age)	Altered bile acid and fatty acid metabolism (e.g., taurochenodeoxycholate-3-sulfate, dicarboxylic acids) and steroid metabolism
Carraro et al. (2018) [40]	Urine	Disruption of tryptophan and fatty acid metabolic pathways
Barlotta et al. (2019) [41]	Urine	Altered tricarboxylic acid cycle metabolites (citrate, isocitrate, oxoglutarate)
Rago et al. (2021) [39]	Blood	Reduced sphingolipid levels (ceramides, sphingomyelins)
Zhu et al. (2022) [42]	Nasopharyngeal samples	High amino acids and low polyunsaturated fatty acids
Raita et al. (2021) [30]	Nasopharyngeal samples	Lipid-dominant metabolic signatures (including sphingolipids and fatty acid metabolites)
Fujiogi et al. (2022) [43]	Nasopharyngeal samples	Reduced sphingolipids and altered phospholipids
Ooka et al. (2022) [45]	Sample Type	Altered lipid metabolism (sphingomyelins, phospholipids, docosapentaenoate)

**Table 2:** VOCs and EBC analysis in children and potential clinical applications.

Research Focus	Biomarker Source	Key Findings	References
Diagnostic	VOCs	Accurate discrimination between asthmatics and healthy controls	van de Kant [56], Smolinska [58], Sas [67]
	EBC	Identification of specific metabolites (purines/leukotrienes)	Esther [52], Montuschi [53], Carraro [59]
Endotyping	VOCs	Differentiation of T2-high vs. Non-Th2 pathways	Meyer [71], Brinkman [74], Schleich [75]
	EBC	Profiling oxidative stress markers for severity stratification	Carraro [54], Fitzpatrick [64]
Correlation with lung function	VOCs	Predicting exacerbations and correlating with airway obstruction	Robroeks [69], Van Vliet [72]
	EBC	Links between pH/metabolites and airway hyperresponsiveness/lung volumes	Kelly [65], van Mastrigt [78]

## Data Availability

No new data were created or analyzed in this study.

## Abbreviations

The following abbreviations are used in this manuscript:

FEV1	Forced Expiratory Volume in 1 s
FEV1/FVC	The ratio of FEV1 to Forced Vital Capacity
FEF25–75%	Forced Expiratory Flow between 25% and 75% of vital capacity
FEFmax	Maximal Forced Expiratory Flow
PEF	Peak Expiratory Flow
VOCs	Volatile Organic Compounds
EBC	Exhaled Breath Condensate
GC-MS	Gas Chromatography–Mass Spectrometry
LC-MS	Liquid Chromatography–Mass Spectrometry
IL	Interleukin
TNF-α	Tumor Necrosis Factor-alpha
IFN-γ	Interferon-gamma
FeNO	Fractional exhaled Nitric Oxide
TSLP	Thymic Stromal Lymphopoietin
GINA	Global Initiative for Asthma
NICE	National Institute for Health and Care Excellence
NMR	Nuclear Magnetic Resonance
PLS-DA	Partial Least-Squares Discriminant Analysis
SERS	Surface-Enhanced Raman Scattering
DNA	Deoxyribonucleic
RNA	Ribonucleic Acid
